# The Relationship Between Pre-Operative Glycosylated Haemoglobin and Opioid Consumption After Caesarean Section in Women With Gestational Diabetes Mellitus

**DOI:** 10.3389/fendo.2022.910914

**Published:** 2022-06-28

**Authors:** Chen Yang, Yue Li, Jianying Hu, Jiangnan Wu, Shaoqiang Huang

**Affiliations:** ^1^ Department of Anaesthesiology, Obstetrics & Gynecology Hospital, Fudan University, Shanghai, China; ^2^ Department of Clinical Epidemiology, Obstetrics & Gynaecology Hospital, Fudan University, Shanghai, China

**Keywords:** gestational diabetes mellitus, postoperative analgesia, cesarean section, visual analog scale, Glycated hemoglobin

## Abstract

**Introduction:**

Women with Gestational diabetes mellitus (GDM) had a higher need and consumption of analgesics than women without GDM. The preoperative level of HbA1c was associated with the postoperative consumption for analgesics in diabetic patients. This prospective observational study go further to investigate the relationship between the pre-operative HbA1c and the post-operative consumption for analgesics in women with GDM.

**Methods:**

Women with GDM and a singleton pregnancy undergoing elective cesarean section under combined spinal-epidural anaesthesia were divided into two groups based on pre-operative HbA1c: group HbA1c < 6% and group HbA1c ≥ 6%. Analgesics consumption, number of patient-controlled analgesia (PCA) compression, and pain scores in 6 hours and 24 hours post-operation were compared between the two groups. Then Pearson’s correlation coefficient and a stepwise multivariate linear regression were performed to investigate possible independentpredictors of post-operative 24-hour sufentanil consumption.

**Results:**

Analgesics consumption was significantly lower (18.8 ± 0.4 vs 23.2 ± 4.3; 82.7 ± 2.4 vs 115.8 ± 17.4, P < 0.001), and number of PCA compressions was significantly less frequent (1 [1-2] vs 3 [1-5]; 5 [3-7] vs 7 [3-15], P < 0.001), and in group HbA1c < 6% than in group HbA1c ≥ 6% in 6 hours and 24 hours post-operation.The univariate analysis showed that sufentanil consumption at 24 hours post-operation was significantly related to pre-operative HbA1c (r = 0.338, P < 0.001) and parity (r = 0.184, P = 0.03) and was related to blood glucose management methods (r = 0.172, P = 0.043). Multivariate linear regression analysis showed that HbA1c was the independent factor related to post-operative 24-hour sufentanil consumption (adjusted r2 = 0.246, P < 0.001)

**Conclusions:**

This study demonstrated that in pregnant women with GDM, the pre-operative HbA1c is independently related to the need for and consumption of analgesics in 24 hours after CS.

## Introduction

Gestational diabetes mellitus (GDM) is a common pregnancy complication. According to the International Diabetes Federation (IDF), the incidence of GDM is approximately 14% worldwide (1). In China, the incidence is 11.91% (2). Unlike classic diabetes mellitus (DM), GDM is a transient form of diabetescharacterized by varying degrees of hyperglycaemia caused by impaired glucose tolerance that is discovered or develops during pregnancy. In most cases, GDM is resolved within one to two months after delivery (3).

Glycosylated haemoglobin (HbA1c) is a highly reliable indicator of blood glucose management in the previous 8 to 12 weeks (4). In a prospective observational study, Kim et al. (5) discovered a positive correlation between perioperative HbA1c and post-operative opioid (fentanyl) consumption in diabetic patients undergoing total hysterectomy. This phenomenon may occur because prolonged hyperglycaemia affects opioid receptors and changes the pharmacokinetics/pharmacodynamics of opioids (6) or because it causes metabolic (7) or neurotransmitter disorders (8). Our previous study (9) showed that immediately after caesarean section (CS), women with GDM had a higher need for and consumption of analgesics than women without GDM. However, we were unable to analyse the relationship between pre-operative HbA1c and post-operative analgesic consumption due to the small size of the GDM group. We hypothesised that there is correlation between pre-operative HbA1c and post-operative opioid consumption.

This prospective observational study enrolled a larger number of women with GDM undergoing CS in order to investigate the ability of pre-operative HbA1c to predict the post-operative need for analgesics and to determine the relationship between pre-operative HbA1c and post-operative opioid consumption.

## Materials and Methods

### Experimental Design

This prospective study was conducted at the Obstetrics and Gynaecology Hospital of Fudan University. Written informed consent was obtained from all subjects participating in the trial. The trial was registered prior to patient enrollment. Women with GDM and a singleton pregnancy who elected to undergo CS under combined spinal-epidural anaesthesia were enrolled. Exclusion criteria were a history of opioid allergy, a history of opioid use, contraindications for spinal anaesthesia, and other pregnancy comorbidities, such as gestational hypertension, gestational hypothyroidism, and pre-eclampsia.

After enrolment, each subject’s medical history was reviewed. Fasting blood glucose (on the morning of surgery), HbA1c, maternal age, height, weight, gestation, CS history, and blood glucose management methods (diet, oral medication, or insulin injections) were recorded.

No pre-medication was given. After the patient entered the operating room, an 18 G trocar needle was used to establish access to a vein in the right upper arm. An in-dwelling urinary catheter was placed. Blood pressure (non-invasive), electrocardiogram (ECG), heart rate, and pulse oximetry were monitored, and baseline values were recorded. The patient was placed in the right decubitus position, a puncture at the L3-4 or L2-3 interspace was performed using the needle-through-needle technique. After the epidural space was identified using the technique of loss of resistance to normal saline, a spinal needle was used to puncture the dura mater and enter the subarachnoid space. Next, 8~10 mg bupivacaine was diluted with cerebrospinal fluid to 3 ml for intrathecal injection, and an epidural catheter was immediately placed. The patient was placed in the supine position, and the operating table was tilted to the left. The sensory block level was tested with a needle every 2 minutes for 10 minutes. The operation was started once the block reached T6. During the operation, 40 µg of phenylephrine was intravenously injected (and the dose was repeated if necessary), and fluid infusion rate was increased in cases of hypotension (systolic blood pressure < 90 mmHg or > 20% below baseline). In cases of sinus bradycardia (heart rate < 50 bpm), 0.2 mg of atropine was intravenously injected, and the dose was repeated if necessary.

Once the infant was delivered and the umbilical cord was clamped, 50 mg of flurbiprofen axetil and 4 mg of ondansetron were intravenously injected (bolus). Before the end of the operation, 5 µg of sufentanil (diluted to 5 ml with normal saline) was injected epidurally. The epidural catheter was then removed. The operation time and blood loss were recorded.

After the operation, the patient was sent to the post-anaesthesia care unit (PACU). An intravenous analgesia pump (Aipeng, Nantong Aipu Medical Equipment Co., Ltd.) was connected once the patient’s blood pressure and heart rate were normal and the block level was below T6. The patient was educated about how to use the pump for patient-controlled intravenous analgesia (PCIA). Analgesics included sufentanil 150 µg and ondansetron 4 mg diluted to 150 ml with normal saline. The background infusion rate was 3 ml/h, the bolus dose was 3 ml, and the locking time was set at 15 minutes. The anaesthesia nurse involved in the study recorded the patients’ use of the post-operative analgesia pump (opioid consumption, number of PCA compression) and any adverse reactions, such as nausea, vomiting, or pruritus. A visual analogue scale (VAS) was used to assess pain at rest and during physical activity at 6 and 24 hours after the operation, with “0 cm” indicating no pain and “10 cm” indicating the worst pain imaginable. Additionally, patient satisfaction with post-operative analgesia was assessed using the following rating scale: 1(very dissatisfied), 2(dissatisfied), 3(neutral), 4 (satisfied), and 5 (very satisfied). Nausea and vomiting were managed with ondansetron 4 mg (intravenous injection), which was repeated if necessary. Patients were excluded from the study under the following conditions: 1) a different anaesthesia method was required due to anaesthesia failure or surgical needs; 2) hysterectomy due to bleeding or other reasons; 3) discontinuation of the use of the analgesia pump for any reason; 4) the patient requested withdrawal from the study.

### Statistical Analysis

SPSS (v 22.0, SPSS, Inc., Chicago IL, USA) was used for the statistical analysis, and P < 0.05 was considered statistically significant. The patients were divided into two groups based on pre-operative HbA1c: group HbA1c < 6% and group HbA1c ≥ 6%. The primary measure was post-operative 24-hour sufentanil consumption. The secondary measures were post-operative 6-hour sufentanil consumption, post-operative 6-hour and 24-hour number of PCA compressions, VAS score, adverse reactions during post-operative analgesia, and patient satisfaction with post-operative analgesia. The Kolmogorov–Smirnov test was performed to confirm whether the data were normally distributed. Normally distributed measurement data were expressed as the mean ± standard deviation (SD) and were analysed with the independent sample t test. Non-normally distributed variables were expressed as median (interquartile range) and were analysed with the Mann-Whitney U test. Categorical variables were expressed as number and were analysed with Fisher’s exact test.

To analyse the relationship between pre-operative HbA1c and post-operative opioid consumption, Pearson’s correlation coefficient was used in the univariate analysis to investigate the relationship between post-operative 24-hour sufentanil consumption and each variable, including fasting blood glucose, HbA1c, gestational age, number of CSs, age, weight, and blood glucose management methods. Then, a stepwise multivariate linear regression was performed to analyse variables with P < 0.2 in the univariate analysis to identify the independent risk factors for post-operative opioid consumption.

The sample size of this study was based on the original hypothesis, assuming a positive correlation between pre-operative HbA1c and post-operative 24-hour sufentanil consumption is 0.4. The enrolment of at least 61 patients was required to allow 90% power to detect a difference between the null hypothesis and the alternative hypothesis using a two-sided hypothesis test with a significance level of P = 0.05. Considering a dropout rate of 10%, it was necessary to enrol at least 69 patients in the study.

## Results

**Figure 1:**
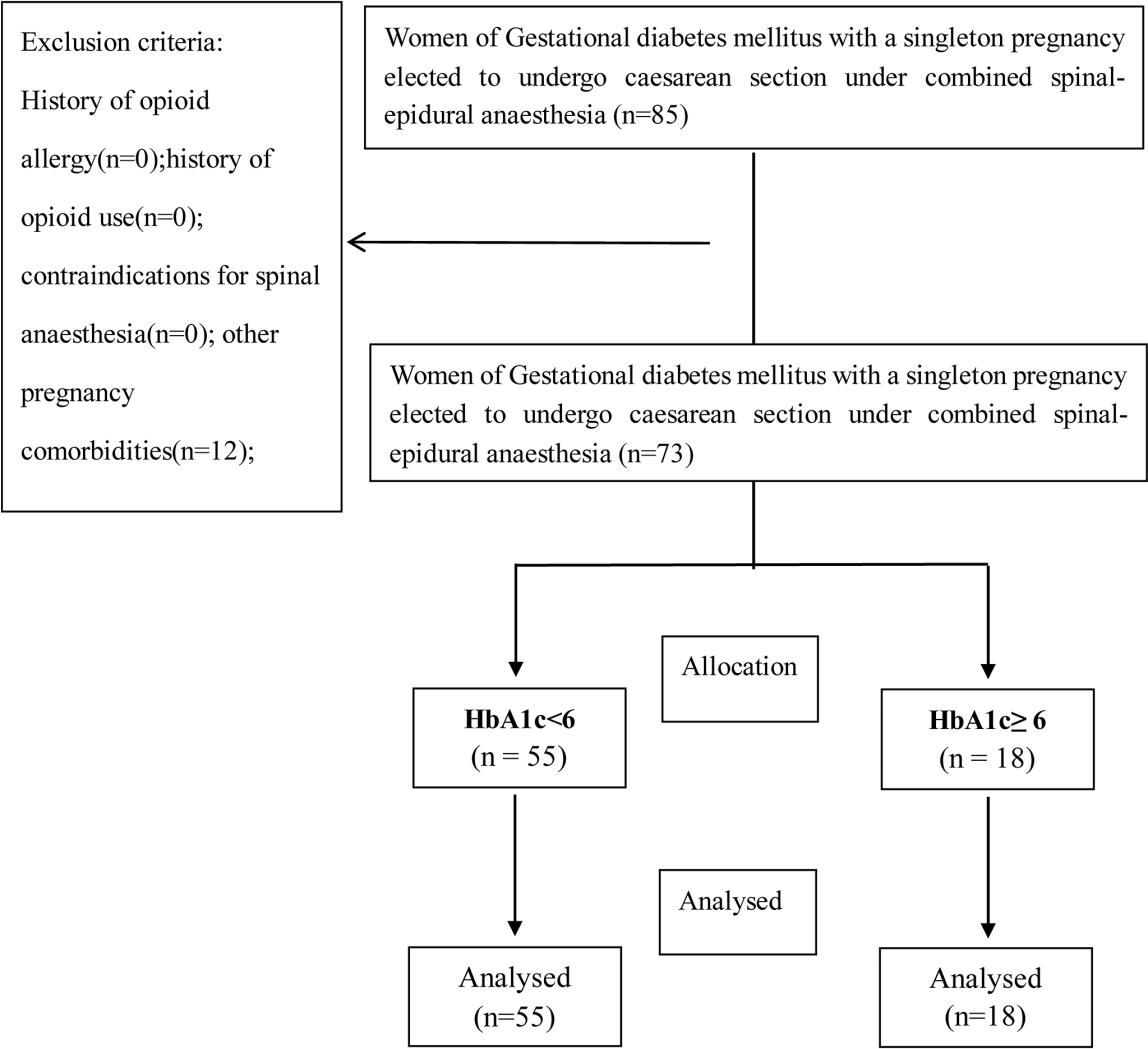
Consort recruitment flow chart.

A total of 73 women with GDM were enrolled in this study, including 55 in group HbA1c < 6% and 18 in group HbA1c ≥ 6%. All the patients completed the study (**Figure 1**). The maternal demographics, intraoperative observations, laboratory tests, and blood glucose management methods (diet management/oral medication/insulin injection) are listed in **Table 1**.

**Table 1:** Clinical characteristics of the patients.

	HbA1c<6 (n=55)	HbA1c≥6 (n=18)	P
**Age (years)**	33.68 ± 1.64	34.04 ± 1.74	0.235
**Gestation (weeks)**	38.14 ± 1.64	37.57 ± 1.11	0.001
**Height (cm)**	162.70 ± 3.20	163.00 ± 5.50	0.56
**Weight (kg)**	72.69 ± 12.15	69.76 ± 10.04	0.08
**Number of CSs (first/repeat)**	39/16	10/8	0.6
**Blood glucose (mmol/l)**	5.58 ± 0.62	5.07 ± 0.67	0.42
**HbA1c (%)**	5.23 ± 0.34	7.29 ± 2.4	0.001
**blood glucose management methods (Diet control/oral drugs/insulin injection)**	47/8/0	6/7/5	0.001
**Amount of bleeding (ml)**	315 ± 65	310 ± 70	0.48
**Duration of surgery (min)**	45. 3 ± 6.7	45.7 ± 5.5	0.56
**Newborn weight(g)**	3262 ± 149.2	3278 ± 147.8	0.34

Data are presented as the mean ± SD or number.

HbA1c was significantly different between the two groups. Group HbA1c ≥ 6% had a smaller gestational age (37.57 ± 1.11 vs 38.14 ± 1.64 weeks, P < 0.001) and a higher rate of insulin use (P < 0.001) than group HbA1c < 6%. The remaining indicators showed no significant differences.

Post-operative analgesia is shown in **Table 2**. Analgesics consumption in 6hours and 24hours post-operation was significantly lower (18.8 ± 0.4 vs 23.2 ± 4.3; 82.7 ± 2.4 vs 115.8 ± 17.4, P < 0.001), and The number of PCA compression in 6 hours and 24 hours post-operation was significantly less frequent (1 [1-2] vs 3 [1-5]; 5 [3-7] vs 7 [3-15], P <0.001), in group HbA1c < 6% than in group HbA1c ≥ 6%. No significant between-group difference was observed in the pain score at any time point (at rest or after physical activity at both 6 and 24 hours after the operation).

**Table 2:** Postoperative analgesia.

		HbA1c<6 (n=55)	HbA1c≥6 (n=18)	P
**Number of PCA** **compression**	6 h	1 [1-2]	3 [1-5]	0.001
	24 h	5 [3-7]	7 [3-15]	0.001
**Sufentanil** **consumption (ug)**	6 h	18.8 ± 0.4	23.2 ± 4.3	0.001
	24 h	82.7 ± 2.4	115.8 ± 17.4	0.001
**VAS scores (cm)** **Rest**	6 h	2 [0-3]	2 [2-2.75]	0.12
	24 h	2 [0-2]	1 [0-2]	0.18
**VAS scores (cm)** **Movement**	6 h	5 [4-6]	5 [5-6]	0.56
	24 h	5 [4-5]	5 [3-5]	0.124

Data are presented as the mean ± SD or median [IQR].

The univariate analysis showed that sufentanil consumption at 24 hours post-operation was significantly related to pre-operative HbA1c (r = 0.338, P < 0.001) and parity (r = 0.184, P = 0.03) and was related to blood glucose management methods (r = 0.172, P = 0.043). The variables with P < 0.2 included age, gestational age, number of CSs, HbA1c, and blood glucose management methods (**Table 3**). Multivariate linear regression analysis showed that HbA1c was the independent factor related to post-operative 24-hour sufentanil consumption (adjusted r2 = 0.246, P < 0.001) (**Table 4**).

**Table 3:** Correlation analysis to examine factors affecting postoperative sufentanyl requirements variable.

Variable	Simplecoefficient	SE	P value	Partial coefficient	P value
**Age (years)**	0.162	0.082	0.057	0.169	0.048
**Weight (kg)**	0.026	0.069	0.758	0.086	0.318
**Number of CSs**	0.184	0.085	0.030	0.262	0.002
**Gestation (weeks)**	-0.128	0.105	0.132	0.012	0.888
**Blood glucose (mmol/l)**	-0.009	0.026	0.916	0.025	0.772
**HbA1c (%)**	0.338	0.053	<0.001	0.339	<0.001
**Blood glucose management methods**	0.172	0.074	0.043	0.065	0.454

**Table 4:** Independent factors affecting postoperative sufentanyl requirements as obtained by multivariate analysis using linear regression with stepwise selection.

Variables	B	SE	95%CI	beta	P	R2	Adjusted R2
**constant**	50.253	10.449	29.586-70.920		<0.001	0.268	0.246
**HbA1c (%)**	2.589	0.62	1.363-3.816	0.33	<0.001		
**Number of CSs**	10.838	2.294		0.391	<0.001		
**Age**	1.176	0.295	0.593-1.758	0.327	<0.001		
**Blood glucose management methods**	4.636	2.078	0.525-8.746	0.183	0.027		

B, regression coefficients; CI, confidence interval; SE, standard error.

No significant difference in side effects or satisfaction with post-operative analgesia was observed between the groups (**Table 5**).

**Table 5:** Comparison of adverse reactions and patient satisfaction between two groups.

	HbA1c<6 (n = 55)	HbA1c≥6 (n = 18)	P
**Nausea**	9	3	0.12
		
**Vomiting**	2	1	0.65
		
**Pruritus**	0	0	0.34
		
**Satisfaction** **(1/2/3/4/5)**	0/0/6/47/2	0/1/3/13/1	0.56
		

Data are presented as number.

## Discussion

This prospective study showed that after CS, women with GDM with pre-operative bad-managed blood glucose had a significantly greater need for and consumption of sufentanil during the first 24 hours post-operation than women with well-managed blood glucose. Post-operative 24-hour sufentanil consumption was related to maternal age, pre-operative HbA1c, the number of CSs, and blood glucose management methods.

Our previous study (9) showed that despite their short duration of high blood glucose, women with GDM had a greater need for opioids after CS. This study (which had a larger sample size in women with GDM than the previous study) showed that for women with GDM, pre-operative HbA1c was an independent risk factor for 24-hour opioid use after CS. HbA1c reflects blood sugar control over a certain period. High blood glucose affects the protein expression levels of opioid receptors (10, 11) and reduces the analgesic potential of opioid receptor agonists (12, 13). Moreover, high blood glucose has pro-inflammatory, pro-oxidative, and pro-thrombotic properties, which may play a key role in enhancing hyperalgesia (14). Ion channels of nociception are upregulated, gamma-aminobutyric acid (GABA)ergic neurons are downregulated, and inhibitory pain signal transmission is weakened, resulting in double hyperalgesia (15). Animal studies have shown that rats with acutely elevated blood glucose for 8 weeks are slow to respond to morphine (16); the short course of GDM may exert similar effects on the body, which may explain why the results of this study are similar to those of DM studies (5, 17).

A retrospective study (18) showed that the need for analgesia was higher in women undergoing a repeated CS than in women undergoing an initial CS. Surgical history is a risk factor for inadequate post-operative analgesia (19). Past surgery often causes severe adhesions, which may contribute to increased post-operative pain (20, 21). Moreover, past surgery may enhance pain sensitivity (18). These data are consistent with our findings that the number of CSs in patients is positively correlated with post-operative 24-hour sufentanil consumption.

A risk factor analysis of GDM based on the International Association of Diabetes Pregnancy Study Groups criteria found that age, history of GDM, family history of diabetes, and large arm circumference are all independent risk factors for GDM (22). Age > 35 years is a risk factor for a high need for analgesia in women with GDM (23). In this study, more women with GDM in group HbA1c ≥ 6% received insulin treatment because treatment for GDM is based on HbA1c. Therefore, age and blood glucose management methods, which are related to HbA1c, are also independent risk factors for post-operative 24-hour sufentanil consumption.

This study has some limitations. First, it followed the internationally accepted diagnostic criteria for GDM (24); that is, for glucose tolerance, the threshold is 5.6 mmol/L for fasting blood glucose, 10.3 mmol/L at 1 hour, 8.6 mmol/L at 2 hours, and 6.7 mmol/L at 3 hours. GDM is confirmed if two or more values meet or exceed the thresholds. No additional tests were performed throughout pregnancy. The patients were instructed to fast for 8-12 hours before the test; however, some (few) patients may not have followed the instructions, resulting in a false positive finding of impaired glucose tolerance. Second, postoperative analgesia did not fully follow the consensus of statement (no use of neutaxial morphine, and NSAIDs was not used enough) (25), we just want to find whether the need for analgesics in patients with GDM is related to HbA1c through intravenous analgesia. We wonder if the effect of different HbA1c levels on the need for analgesics still exists after the application of neuraxial morphine and regular administration of sufficient NSAIDs. Finally, this study enrolled only patients with simple GDM. Patients with other pregnancy comorbidities, such as thyroid dysfunction during pregnancy (26), which may be related to the development of GDM, were excluded from this study to minimize interference. Therefore, the results of this study apply only to women with simple GDM post-operation, and further studies are needed to investigate the need for long-term post-operative analgesia and the analgesic needs of patients with multiple pregnancy comorbidities.

## Conclusion

For women with GDM, pre-operative HbA1c is independently related to the need for and consumption of analgesics during the 24 hours after CS. HbA1c should be closely monitored in women with GDM and advanced maternal age or a history of CS to provide personalized treatment and improve the quality of and satisfaction with post-operative analgesia.

## Data Availability Statement

The original contributions presented in the study are included in the article/supplementary materials. Further inquiries can be directed to the corresponding author.

## Ethics Statement

The studies involving human participants were reviewed and approved by the Ethics Committee of the Obstetrics and Gynaecology Hospital of Fudan University. The patients/participants provided their written informed consent to participate in this study.

## Author Contributions

CY and SH conceived the study. JW conducted the statistical analyses. CY, YL, and JH collected and interpreted the clinical data. CY and SH drafted the manuscript. All authors contributed to the interpretation of the results and approved the final manuscript.

## Conflict of Interest

The authors declare that the research was conducted in the absence of any commercial or financial relationships that could be construed as a potential conflict of interest.

## Publisher’s Note

All claims expressed in this article are solely those of the authors and do not necessarily represent those of their affiliated organizations, or those of the publisher, the editors and the reviewers. Any product that may be evaluated in this article, or claim that may be made by its manufacturer, is not guaranteed or endorsed by the publisher.

## Abbreviations

ASA, American Society of Anesthesiologists; GDM, Gestational diabetes mellitus; HbA1c, Glycated hemoglobin; NGDM, Non-GDM; PCA, Patientcontrolled analgesia; PCIA, Patient-controlled intravenous analgesia; SPSS, Statistical package for social science; VAS, Visual analog scale.
